# Mouse models to decipher anti-tumor immunity

**DOI:** 10.18632/oncotarget.27111

**Published:** 2019-08-20

**Authors:** Thomas Parigger, Richard Greil, Nadja Zaborsky

**Affiliations:** Department of Internal Medicine III with Haematology, Medical Oncology, Haemostaseology, Infectiology and Rheumatology, Oncologic Center, Salzburg Cancer Research Institute – Laboratory for Immunological and Molecular Cancer Research (SCRI-LIMCR), Paracelsus Medical University, Salzburg, Austria; Cancer Cluster Salzburg, Austria

**Keywords:** CLL mouse model, genetic heterogeneity, clonal evolution, immune oncology

**Figure 1:**
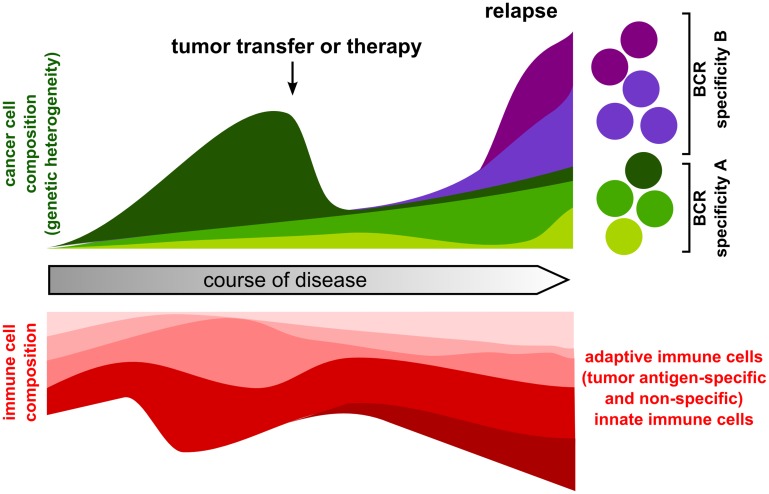
Coevolution of cancer cells and immune cells over time.

Next generation sequencing has provided important insight into genome dynamics during cancer progression, reflected in consecutive acquisition of mutations and chromosomal aberrations and expansion of particular cancer subclones based on a distinctive genomic profile [1]. Alongside this remarkable molecular plasticity, significant changes within the tumor microenvironment and immune cell composition have been noticed, revealing fundamental cancer-immune cell crosstalk [2]. These analyses yield important information on factors decisive for how cancer overcomes immune surveillance and on factors contributing to (immune) therapy resistance. In this regard, chronic lymphocytic leukemia (CLL) has become an interesting model disease to study cancer-immune cell coevolution, because CLL has a long latency phase, allowing longitudinal analysis (including consecutive therapies) and additionally, CLL is amenable to high purity cancer sampling and immune phenotyping from simple blood draws [3]. However, complementing to studies on human samples, mouse models provide a unique opportunity to interrogate complex cancer-immune interactions much more straight forward: other organs (lymphoid and non-lymphoid) apart from peripheral blood, are easily accessible, the genetic background of mice including the major histocompatibility haplotype is standardized and mice -including their immune cells- are genetically manipulable. However, to reasonably exploit these advantages, it is important to know whether a particular mouse model is genetically and immune-phenotypically similar to the human disease. In this regard, it is conceivable that cancer development based on transgene overexpression not automatically entails genetic heterogeneity but may render additional driver mutations unnecessary. In a recent study, we analyzed the genetic landscape in a widely used mouse model for CLL, the TCL1 mouse [4]. In this mouse, the human TCL1 transgene is expressed specifically in B cells, leading to development of a CLL-like disease, typically featuring a long latency phase followed by expansion of CD5 CD19 double positive B cells in peripheral blood with lymph node infiltrations [5]. Similar to human CLL, the TCL1 mouse develops deficits in adaptive immunity such as CD4/CD8 T cell skewing, increased T cell exhaustion and impaired immune synapse formation (reviewed in [6]). In our study, we found that the TCL1 mouse similar to human CLL has high inter- and intratumor genetic heterogeneity, revealing that in addition to TCL1 transgene expression, additional genetic events are necessary for tumor development. The identified mutations mapped to biological pathways, which were previously described to be implicated in human CLL, indicating similar dependency on core signals that contribute to CLL growth and survival when comparing human and murine CLL [7, 8]. Most strikingly, our data further showed that clonal evolution of leukemic cells in mice is extremely dynamic, particularly upon transfer of primary tumors into syngeneic wildtype recipient mice. This plasticity is not only based on the occurrence of novel subclonal somatic mutations but also on a high initial B cell receptor (BCR) specific heterogeneity. This means, that although most of the leukemic cells of a particular CLL case have the same BCR specificity, there are many minor clones with a distinctive BCR profile and hence, also with a distinctive somatic mutation landscape. These minor clones can dramatically expand upon transfer. Thus, interrogating genetic landscapes of TCL1 tumors alongside immune phenotyping in wildtype, immune compromised or genetically modified hosts or in mice subjected to preclinical treatment studies will definitely yield important insight into cancer-immune/microenvironment crosstalk during disease progression and treatment response (Figure 1).
